# Daily Inhaled Corticosteroids Treatment Abolishes Airway Hyperresponsiveness to Mannitol in Defence and Police Recruits

**DOI:** 10.3389/falgy.2022.864890

**Published:** 2022-05-31

**Authors:** Clair D. Lake, Keith K. H. Wong, Clare P. Perry, Heikki O. Koskela, John D. Brannan

**Affiliations:** ^1^Department of Respiratory and Sleep Medicine, Royal Prince Alfred Hospital, Camperdown, NSW, Australia; ^2^Faculty of Medicine and Health, University of Sydney, Camperdown, NSW, Australia; ^3^Woolcock Institute of Medical Research, Glebe, NSW, Australia; ^4^Department of Respiratory and Sleep Medicine, Westmead Hospital, Westmead, NSW, Australia; ^5^Unit for Medicine and Clinical Research, Pulmonary Division, Kuopio University Hospital, Kuopio, Finland; ^6^Faculty of Health Sciences, School of Medicine, Institute of Clinical Sciences, University of Eastern Finland, Kuopio, Finland; ^7^Department of Respiratory and Sleep Medicine, John Hunter Hospital, New Lambton, NSW, Australia; ^8^School of Medicine and Public Health, University of Newcastle, Callaghan, NSW, Australia

**Keywords:** airway hyperresponsiveness (AHR), asthma, inhaled corticosteroids, indirect bronchial challenge test, occupational assessment

## Abstract

**Background:**

Airway hyperresponsiveness (AHR) is a key pathophysiological feature of asthma and causes exercise-induced bronchoconstriction (EIB). Indirect bronchial provocation tests (BPTs) (e.g., exercise, mannitol) aid to diagnose asthma and identify EIB. Daily inhaled corticosteroids (ICS) can abolish AHR caused by indirect stimuli. Where strenuous physical exertion is integral to an occupation, identification of those at risk of EIB is important and documentation of inhibition of AHR with ICS is required before recruitment.

**Methods/Objectives:**

A retrospective analysis was performed on 155 potential recruits with AHR to mannitol who underwent follow-up assessment after daily ICS treatment to determine the proportion that can abolish AHR using ICS and to determine any predictors of the persistence of AHR.

**Results:**

Airway hyperresponsiveness was abolished in the majority (84%, *n* = 130) over the treatment period (mean ± SD 143 ± 72days), and it was defined as the provoking dose of mannitol to cause a 15% fall in FEV1 (cumulative inhaled dose of mannitol to cause 15% fall in FEV_1_, PD_15_) improved from (GeoMean) 183 to 521 mg. Compared with recruits in whom AHR was abolished with daily ICS (i.e., no 15% fall in FEV_1_ to the maximum cumulative dose of mannitol of 635 mg), in those where AHR remained (16%, *n* = 25), baseline AHR was more severe (PD15: 85 mg vs. 213 mg, *P* < 0.001), baseline FEV_1_% was lower (89 vs. 96%; 95%CI:2–12, *P*=0.004), and they had a longer follow-up duration (180 vs. 136 days; 13–74, *P* = 0.006). Baseline FEV_1_% (adjusted odds ratio 0.85; 95%CI:0.77–0.93), FEV_1_/FVC (0.78; 0.67–0.90), FEF_25−75%_ (1.15; 1.06–1.25), and airway reactivity to mannitol (%Fall/cumulative dose of mannitol multiplied by 100) (1.07; 1.03–1.11) predicted AHR remaining after daily ICS.

**Conclusion:**

Airway hyperresponsiveness to mannitol can be abolished after 20 weeks of daily treatment with ICS. Inhibition of AHR is likely due to attenuation of airway inflammation in response to ICS treatment. Increased airway reactivity and lower spirometry variables predicted the persistence of AHR. Thus, those with a slower response to daily ICS on AHR can potentially be identified at the commencement of monitoring ICS using inhaled mannitol.

## Plain Language Summary

Airway hyperresponsiveness (AHR) is a key feature of asthma. In certain occupational settings, such as the military, AHR to mannitol is used to assess asthma and the risk of exercise induced-bronchoconstriction (EIB). Reduced airway calibre in the form of lower normal spirometry and more significant AHR (moderate to severe AHR to mannitol) predict a slower response to ICS treatment if the goal is to abolish AHR. Therefore, assessing AHR to mannitol along with spirometry prior to treatment may be useful to help predict the speed one can benefit from using daily ICS therapy, and the likelihood to abolish AHR.

## Introduction

Airway hyperresponsiveness (AHR) is a key pathophysiological feature of asthma and is responsible for exercise-induced bronchoconstriction (EIB) (1). EIB commonly occurs in those where asthma is active and is characterised by bronchospasm following vigorous exercise. Indirect bronchial provocation tests (BPT) (e.g., exercise, eucapnic voluntary hyperpnoea, inhaled mannitol) can be performed to diagnose asthma and to identify EIB (2). Indirect BPT provokes bronchoconstriction by causing a release of inflammatory mediators from mast cells and eosinophils in the airways (2). Daily treatment with inhaled corticosteroids (ICS) decreases the number of inflammatory cells in the airway (3). AHR to mannitol can be attenuated using daily ICS treatment over 6–9 weeks of therapy (4). A reduction in the number of inflammatory cells may be reflected by attenuation of AHR, therefore AHR may be useful to monitor therapeutic response to daily ICS (5). Loss of AHR may also be a potential surrogate marker for EIB inhibition following daily treatment with ICS. The optimal goal of treatment when using indirect BPT to monitor the therapeutic response of using ICS treatment is to abolish AHR (6). This is particularly relevant for people with asthma or those with EIB who may work in occupations that involve vigorous exercise, thus EIB needs to be inhibited (7).

Strenuous physical exertion is integral in occupations such as in the military, police, and firefighters. Screening of individuals prior to recruitment into such professions using indirect BPT has been shown to be useful to identify those with AHR and at risk of EIB (8, 9). AHR may prevent optimal exercise performance, require the need for acute rescue therapy, and in very rare cases induce a fatal attack of asthma (10). Due to these risks associated with AHR, the objective identification of AHR using indirect BPT is required by both the Australian Defence Force (ADF) and the New South Wales Police Force (NSWPF). Candidates or “recruits” applying for positions in the ADF and NSWPF with a history of asthma are referred to pulmonary function laboratories (PFL) as part of a medical assessment for investigation of possible AHR using the mannitol BPT. Where significant AHR is demonstrated, ICS naïve recruits are prescribed ICS either alone or in combination with a long-acting beta-agonist (LABA). Any recruits already taking ICS have their dose of ICS either maintained or adjusted. Recruits with asthma may be accepted into the ADF and NSWPF if they can demonstrate resolution of AHR to mannitol following daily treatment with ICS. However, while AHR can be attenuated in some individuals, AHR can persist despite therapy. It is not known if there are characteristics that predict the persistence of AHR to mannitol.

We performed a retrospective analysis of occupational recruits having AHR to mannitol monitored in the presence of daily ICS. We also determined the proportions of recruits in whom AHR to mannitol was abolished and if baseline characteristics might determine persistence of AHR.

## Methods

### Participants

Data were only included from individuals seeking employment (i.e., recruits) in the ADF or NSWPF. Data were included in the analysis where an initial mannitol BPT was positive and where reassessment of mannitol BPT occurred on one or more occasions within a 12-month period while using daily treatment with ICS. The majority of participants were talking low dose ICS which was a part of the ADF recruitment policy. This is not the policy of the NSWPF where standard doses of ICS in combination with a LABA can be used. Where more than one reassessment occurred within a 12-month period, the follow-up data were reported from the visit with the longest duration. Data were excluded where the reassessment period was >12-months or ICS treatment was not used.

### Study Design

A retrospective review of the patient databases of the PFL at Westmead and Royal Prince Alfred Hospitals from 2005 to 2018 was performed. A review of each database was approved by the Western Sydney and Sydney Local Health District Human Research Ethics Committees, respectively.

### Spirometry and Mannitol Bronchial Provocation Test

Spirometry was performed immediately before the mannitol BPT according to American Thoracic Society and European Respiratory Society criteria (11), and predicted values were determined by comparison to reference values (12). The mannitol BPT (Aridol®/Osmohale® Pharmaxis Ltd, Frenchs Forest, NSW, Australia) was performed according to the standardised test protocol (13).

The provoking dose of mannitol causing a 15% fall in FEV_1_ (PD_15_) is a measure of airway sensitivity to mannitol. The response is classified as severe if the PD_15_ is ≤35 mg, moderate if >35 mg to ≤155 mg, and mild if >155 mg to 635 mg. If the fall in FEV_1_ is <15% (no PD_15_), then the test is negative, indicating the absence of AHR to mannitol.

Airway sensitivity to mannitol can also be determined using the dose that provokes a fall in FEV_1_ of 10% (PD_10_). The PD_10_ is associated with mild EIB and therefore the PD_10_ was also calculated for those that had a final fall in FEV_1_ of between 10 and 15% (14, 15).

The degree of airway reactivity to mannitol (percent fall in FEV_1_ divided by the final cumulative dose of mannitol), known as the response dose ratio (RDR), was also assessed. To simplify interpretation, the RDR was multiplied by 100 (RDR_100_). Complete resolution of AHR would be considered to have been achieved following effective ICS treatment if the RDR_100_ < 1, as this threshold represents the upper limit of reactivity and a flat dose response curve that has been observed in healthy non-asthmatic subjects (16).

A *post hoc* subgroup analysis was performed to assess the response to ICS treatment in recruits with moderate to severe AHR to mannitol at baseline (PD_15_ ≤155 mg), as this degree of AHR has been shown to be associated with a greater degree of airway inflammation (17, 18). We did this in order to determine if the degree of AHR to mannitol influences response to ICS treatment.

### Statistics

Descriptive summaries and spirometry data are reported as mean and SD or number and percentage of the group. PD_15_, PD_10_, and RDR_100_ results were log transformed (base 2) for analysis and presented as the geometric mean (Gmean) and 95% CI. Where a 0% fall in FEV_1_ occurred, a fall of 0.1% was imputed to enable the calculation of RDR_100_. A PD_15_ of 635 mg was imputed when a PD_15_ did not occur on follow-up (interpreted as the resolution of AHR). Groups were generated based on follow-up PD_15_ (AHR abolished, noPD_15_ vs. persistent AHR, PD_15_ < 635 mg), follow-up PD_10_ (noPD_10_ vs. PD_10_), and RDR_100_ at follow up (<1 vs. >1). Independent samples *t*-tests were used to assess differences between groups. Paired samples *t*-tests were used for within group change from baseline to the follow-up visit. Spearman's correlation was used to assess relationships between variables at baseline and at follow-up and to assess relationships between the improvements in variables at the follow-up visit. Chi-squared and Fishers exact tests were used to compare proportions within and between groups.

Receiver operating characteristic (ROC) curves were created for baseline variables (PD_15_, RDR_100_, FEV_1_ %predicted, FEV_1_/FVC%, and FEF_25−75%_ %predicted) to evaluate potential predictors of persistent AHR at follow up ICS treatment visit. The area under the curve (AUC) was calculated and “optimal” thresholds were reported, corresponding to the point on the ROC curve closest to the upper left corner. To generate a prediction model for AHR remaining on follow-up, the first univariate binary logistic regression was performed on baseline variables to determine potential predictors. Variables suggestive of an association (*P* < 0.1) with persistent AHR were considered for multivariate logistic regression analysis, performed on these variables with a backward stepwise process.

SPSS statistics version 26 was used for statistical analysis (IBM, Chicago, IL, USA).

## Results

A total of 155 recruits with at least two visits over an average of 143 days [20.4 weeks] were analysed (Table 1). The majority were male [83%], in early adulthood [22 years ± 5], and were seeking employment in the ADF [72%] as opposed to applying for the NSWPF. Only one quarter [24%] were taking ICS prior to their baseline visit (Table 1). During the follow-up period, the majority were taking ICS alone [86%] with the remainder taking ICS with LABA in combination. A sub-analysis of those taking LABA in combination with ICS vs ICS alone found there were no differences in demographics or lung function characteristics between the groups and this also included no differences in AHR. However the numbers taking combination LABA/ICS (24%) were lower than those taking ICS alone (86%) (Table 1). At the baseline visit, group mean values for spirometry [FEV_1_ 95% predicted ± 11] were within normal reference limits and the geometric mean (Gmean) PD_15_ represented mild AHR [Gmean (95%CI) PD_15_ 184 mg (159 to 212)] (Table 1).

**Table 1:** Total cohort (*n* = 155): Baseline and ICS follow up: Demographics, lung function, AHR severity, ICS therapy at baseline visit, ICS type and duration of follow up period.

	**Baseline**	**Follow up**	**Difference (95%CI)**	***P***
Demographics				
Age (years), mean SD	22 ± 5			
Male, *n*%	128 (83)			
BMI, kg/m^2^, mean SD	25 ± 4			
Lung function				
FEV_1_, % predicted, mean SD	95 ± 11	97 ± 12	2 (1 to 3)	**<0.001**
FEV_1_/FVC%, mean SD	78 ± 8	80 ± 8	2 (1 to 6)	**<0.001**
FEV_1_/FVC (<LLN), *n*%	39 (25)			
FEF_25−75%_ % predicted, mean SD	77 ± 21	83 ± 23	6 (4 to 9)	**<0.001**
FEF_25−75%_ (<65% predicted), *n*%	47 (30)			
AHR to mannitol				
PD_15_, mg, Gmean (95%CI)	184 (159 to 212)	521 (482 to 564)		**<0.001**
Moderate to Severe AHR (PD_15_ < 155 mg), *n*%	61 (39)			
RDR_100_, %/mg, Gmean (95%CI)	7.6 (6.6 to 8.7)	1.3 (1.1 to 1.5)		**<0.001**
PD_15_, fold increase, Gmean (95%CI)		2.8 (2.5 to 3.2)		**<0.001**
RDR_100_, fold decrease, Gmean (95%CI)		5.9 (4.9 to 7.1)		**<0.001**
PD_15_, doubling dose, Gmean (95%CI)		1.5 (1.3 to −1.7)		**<0.001**
ICS Treatment				
ICS use at baseline, *n*%	37 (24)			
ICS alone, *n*%		133 (86)		
Follow-up duration, days, mean SD		143 ± 72		

*Values are mean ± SD or number (% of group). PD_15_ and RDR_100_ Geometric mean (Gmean) 95%CI, logged for analysis. AHR, airway hyperresponsiveness; PD_15_, provocative dose of mannitol causing a 15% fall in FEV_1_; RDR_100_, Response dose ratio; FEF_25−75%_, forced expiratory flow _25−75%_ %predicted; ICS, Inhaled corticosteroids. Bold values that indicate statistical significance*.

### Resolution of AHR to Mannitol

**Figure 1:**
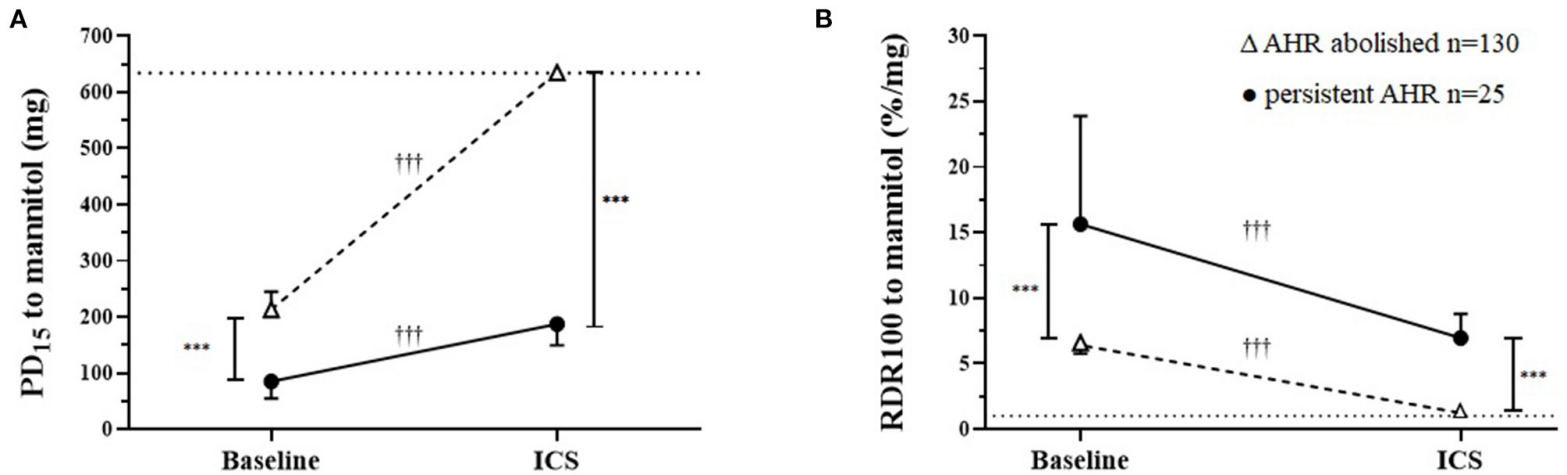
Airway sensitivity and reactivity to mannitol at baseline and after a mean of 20 weeks daily inhaled corticosteroids (ICS) in those where airway hyperresponsiveness (AHR) was either abolished or was persistent. **(A)** Cumulative inhaled provoking dose of mannitol to cause 15% fall in FEV_1_ (PD_15_) baseline and follow-up ICS treatment geometric mean and 95% CI. The dotted horizontal line represents the total cumulative dose (635 mg) the upper limit indicating the mildest AHR. **(B)** Baseline and follow-up ICS treatment for response dose ratio (RDR_100_) geometric mean and 95% CI. The dashed line represents the mean response for healthy non-asthmatics who do not have AHR to mannitol. ^*†*^Within/ ^*^Between group differences *P*-value: <0.05^*^^†^, <0.1^**^^††^, <0.001^***^^†††^.

Resolution of AHR to mannitol (i.e., noPD_15_) was achieved in most recruits [*n* = 130/155 (84%)] with a mean follow-up period of 136 days (Table 2; Figure 1). For the total group, with daily treatment with ICS, there was a 3.0-fold increase in PD_15_ (95%CI 2.6 to 3.4) equating to an increase of 1.6 doubling doses (DD) (95%CI: 1.4 to 1.8) (Table 1). However, nearly one-third [*n* = 42/130 (32%)] of those that abolished their PD_15_ had a PD_10_ at the follow-up visit (Table 3). Over one-third [*n* = 56 (36%)] of all recruits showed complete suppression of the airway response to mannitol or a flat dose response curve, defined by an RDR_100_ <1 at the end of the follow-up period (i.e., these subjects neither had a PD_10_ nor PD_15_) (Table 4).

**Table 2:** Baseline demographics, spirometry, airway sensitivity and reactivity ICS therapy at baseline visit, ICS type and duration of follow up period; Grouped based on airway sensitivity (PD_15_) on final ICS treatment visit.

	**Airway sensitivity (PD**_**15**_ **) at follow up**			***P***
	**Persistent PD15**	**Abolished PD15**	**Difference (95%CI)**	
Demographics				
Total *n*	25	130		
Age, years, mean (SD)	24 ± 8	21 ± 5	2 (−0.04 to 5)	0.054
Males, *n* (%)	21 (84)	107 (82)	2 (0.2 to 3)	1
BMI, kg/m^2^, mean SD	25 ± 5	25 ± 3	0.3 (−1 to 2)	0.74
Spirometry				
FEV_1_, % predicted, mean (SD)	87 ± 13	96 ± 11	7 (2 to 12)	**<0.01**
FEV_1_/FVC%, mean (SD)	74 ± 9	78 ± 8	4 (1 to 8)	**0.01**
FEF_25−75%_, % predicted, mean (SD)	69 ± 25	78 ± 20	10 (1 to 19)	**0.03**
Baseline FEF_25−75%_ <65% predicted, *n* (%)	12 (48)	27 (21)	27 (4 to 50)	**<0.01**
Baseline FEV_1_/FVC <LLN, *n* (%)	15 ± 60	32 (74)	14 (12 to 58)	**<0.001**
AHR to mannitol				
PD_15_, mg, Gmean (95%CI)	85 (55 to 132)	213 (186 to 245)		**<0.001**
Mod-Sev AHR (PD_15_ < 155 mg), *n* (%)	18 (72)	43 (33)	39 (17 to 61)	**<0.001**
RDR_100_, %/mg, Gmean (95%CI)	27.2 (36.6)	9.5 (10.3)		**<0.001**
ICS Treatment				
ICS use at baseline, *n* (%)	11 (44)	26 (20)		**<0.01**
ICS alone at follow-up, *n* (%)	21 (84)	112 (86)		0.78
Follow-up duration, days, mean (SD)	180 ±83	136 ± 68	43 (13 to 74)	**<0.01**

*Values are mean ± SD or number (% of group). χ^2^ analysis used for proportions; Fisher exact used where estimated counts <5. PD_15_ and RDR_100_ Geometric mean (Gmean) 95%CI, logged for analysis. Bold values that indicate statistical significance*.

**Table 3:** Recruits baseline demographics, spirometry, AHR and ICS treatment; Grouped based on airway sensitivity (PD_10_) at follow-up ICS treatment visit.

	**Airway sensitivity on follow up (PD**_**10**_ **)**			***P***
	**Persistent PD10**	**Abolished PD10**	**Difference (95%CI)**	
Demographics				
Total group proportion, *n* (%)	67 (43)	88 (57)		
Age, years, mean (SD)	22 (6)	22 (5)	0.2 (-2 to 2)	0.84
Males, *n* (%)	53 (79)	75 (85)	6 (-20 to 7)	0.43
Spirometry				
FEV_1_, % predicted, mean (SD)	90 (11)	98 (11)	8 (4 to 11)	**<0.001**
FEV_1_/FVC%, mean (SD)	75 (8)	79 (8)	4 (1 to 6)	**0.004**
FEF_25−75%_, % predicted, mean (SD)	70 (19)	82 (21)	12 (5 to 18)	**<0.001**
FEF_25−75%_ <65% predicted, *n* (%)	25 (37)	8 (9)	28 (14 to 43)	**<0.001**
FEV_1_/FVC <LLN, *n* (%)	23 (34)	16 (18)	16 (1 to 31)	**0.04**
AHR to mannitol				
PD_15_, mg, Gmean (95%CI)	130 (100 to 168)	239 (208 to 276)		**<0.001**
Moderate-Severe AHR (PD_15_ <155mg), *n* (%)	38 (57)	23 (26)	31 (14 to 47)	**<0.001**
RDR_100_, %/mg, Gmean (95%CI)	10.7 (8.3 to 13.7)	5.9 (5.1 to 6.7)		**<0.001**
ICS Treatment				
ICS use at baseline, *n* (%)	21 (31)	16 (18)		0.09
ICS alone, *n* (%)	57 (85)	76 (86)		1
Follow duration, days, mean (SD)	151 (80)	138 (65)	−13 (−36 to 10)	0.26

*Values are mean ± SD or number (% of group). χ^2^ analysis used for proportions. PD_15_ and RDR_100_ Geometric mean, logged for analysis. Bold values that indicate statistical significance*.

**Table 4:** Recruits baseline demographics, spirometry, AHR and ICS treatment; Grouped based on airway reactivity (RDR_100_) at follow-up ICS treatment visit.

	**Airway reactivity on follow up (RDR**_**100**_ **)**			***P***
	**RDR_**100**_ < 1**	**RDR_**100**_ > 1**	**Difference (95%CI)**	
Demographics				
Total group proportion, *n* (%)	56 (36)	99 (64)		
Age, years, mean (SD)	22 (6)	22 (5)	−0.1 (−2 to 2)	0.89
Males, *n* (%)	44 (79)	84 (85)	6 (−20 to 8)	0.44
Spirometry				
FEV_1_, % predicted, mean (SD)	98 (11)	92 (11)	−6 (−10 to −2)	**0.001**
FEV_1_/FVC%, mean (SD)	80 (8)	76 (98)	−4 (−6 to −1)	**0.008**
FEF_25−75%_, % predicted, mean (SD)	83 (22)	73 (20)	−10 (−17 to −4)	**0.003**
FEF_25−75%_ < 65% predicted, n (%)	12 (21)	35 (35)	14 (−30 to 2)	0.10
FEV_1_/FVC < LLN, *n* (%)	10 (18)	29 (29)	11 (−26 to 3)	0.17
AHR to mannitol				
PD_15_, mg, Gmean (95%CI)	209 (174 to 250)	171 (140 to 209)		0.19
Moderate-Severe AHR (PD_15_ < 155 mg), *n* (%)	18 (32)	43 (43)	11 (−28 to 6)	0.23
RDR_100_, %/mg, Gmean (95%CI)	6.5 (5.5 to 7.8)	8.3 (6.8 to 10.1)		0.11
ICS Treatment				
ICS use at baseline, *n* (%)	10 (18)	27 (27)		0.26
ICS alone, *n* (%)	43 (77)	90 (91)		**<0.001**
Follow duration, days, mean (SD)	138 (55)	147 (80)	−8 (−33 to 15)	0.46

*Values are mean ± SD or number (% of group). χ^2^ analysis used for proportions. PD_15_ and RDR_100_ Geometric mean (Gmean), logged for analysis. Bold values that indicate statistical significance*.

### Persistent AHR to Mannitol

**Figure 2:**
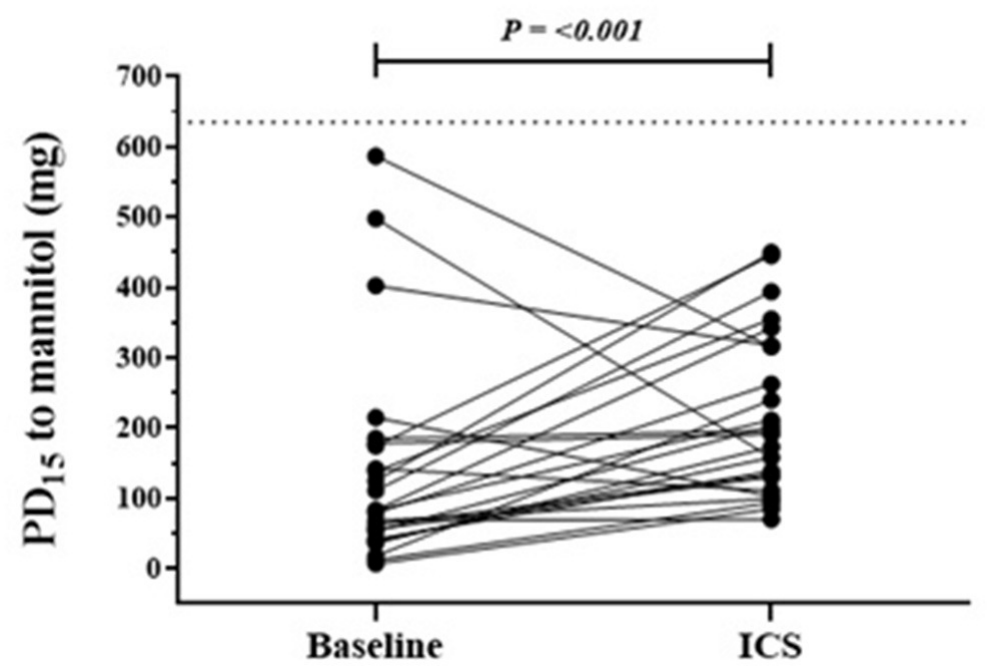
Individual response in those with persistent AHR to mannitol following a mean of 20 weeks of daily ICS. Baseline and follow-up individual PD_15_ to mannitol results in the group where AHR remained at ICS follow-up visit. *n* = 25, follow up mean duration 180 days. The dotted horizontal line represents the total cumulative dose (635 mg) of inhaled mannitol during bronchial provocation and the upper limit indicates the mildest AHR.

In the group for whom AHR persisted in the presence of daily ICS, the mean baseline AHR severity was in the moderate range [PD_15_ Gmean (95% CI) 85 mg (55 to 132)], and these subjects still showed modest but statistically significant improvements in AHR [PD_15_ 2.2-fold increase, *P* < 0.001] with ICS treatment (Table 2; Figure 2). These recruits were also more likely to be taking ICS prior to occupational assessment and were on ICS therapy for a mean of 43 days longer during the assessment compared with recruits in whom AHR was abolished (Table 2). In the group where AHR persisted, there was a greater proportion of individuals with an abnormal FEF_25−75%_, FEV_1_/FVC below the lower limit of normal (LLN), and moderate to severe AHR (PD_15_ <155 mg) at the baseline visit (Table 2).

### Response to Daily Treatment With ICS–Spirometry

**Figure 3:**
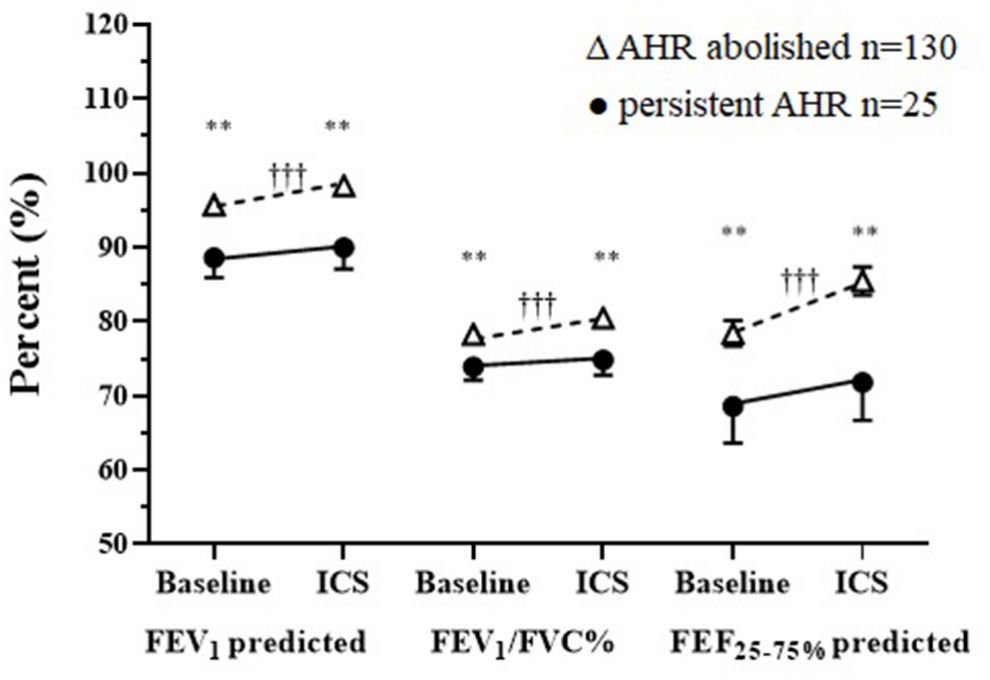
Baseline and follow-up treatment spirometry results following a mean of 20 weeks daily ICS in those where AHR was either abolished or was persistent for FEV_1_ and FEF_25−75%_ % predicted, FEV_1_/FVC absolute % mean ± SD. †Within/*Between group differences at each visit. *P*-value: <0.05* ^*†*^, <0.1** ^*††*^, <0.001*** ^*†††*^.

There were improvements in spirometry with significant changes in FEV_1_, FEV_1_/FVC, and FEF_25−75%_ following daily treatment with ICS over the assessment period for the total group (Table 1). Although the mean baseline spirometry values for both groups (PD_15_ and noPD_15_) were within normal reference limits, statistically significant improvements were observed in all spirometry variables in the group in which AHR was abolished (Figure 3). In contrast, there were no improvements in any spirometry variables in the group whose AHR persisted.

### Association Between Spirometry Variables and AHR to Mannitol

There was a very weak but statistically significant relationship between baseline PD_15_ and FEV_1_ and with FEF_25−75%_ (Table 5). There was a weak but significant relationship between the improvement in RDR_100_ with the improvement in FEV_1_, FEV_1_/FVC, and FEF_25−75%_ (Table 5).

**Table 5:** Spearman's correlation between change in spirometry and change in RDR_100_ from baseline to follow-up ICS treatment visit and between baseline PD_15_ and baseline spirometry variables.

		**Rs**	***P***
Baseline PD_15_	Baseline		
	FEV_1_ % predicted	0.164	**0.04**
	FEF_25−75%_ % predicted	0.160	**0.05**
Δ RDR_100_	Δ FEV_1_ % predicted	0.350	**<0.001**
	Δ FEV_1_/FVC	0.367	**<0.001**
	Δ FEF_25−75%_ % predicted	0.419	**<0.001**

Δ: *Change. Rs: Spearman's correlation coefficient. Bold values that indicate statistical significance*.

### Predictors of Persistent AHR to Mannitol

Receiver operating characteristic analysis was used to evaluate the baseline lung function variables as predictors of persistent PD_15_ with ICS treatment. The AUC for baseline measurements of FEV_1_, FEV_1_/FVC, FEF_25−75%_, PD_15_, and RDR_100_ were 0.68, 0.66, 0.67, 0.75 and 0.76, respectively. The optimal thresholds that predicted AHR persisting in this population were <91% for FEV_1_, <75% for FEV_1_/FVC, <64% for FEF_25−75%_, <142 mg for PD_15_, and >8.6%/mg for RDR_100_.

**Figure 4:**
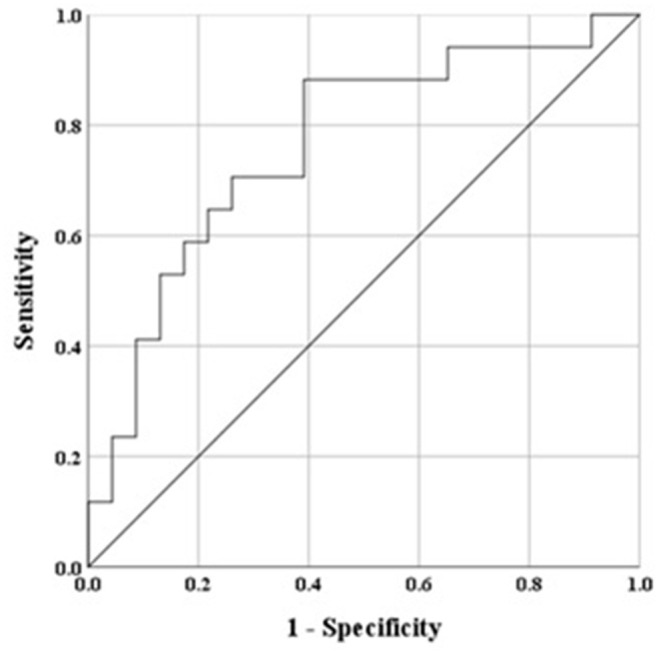
Receiver operator characteristic (ROC) based on baseline variables to predict persistent AHR to mannitol, ROC curve final multivariate model, area under the curve (AUC) for the final model was 0.851, whereby a value of 0.5 indicates chance model performance and 1.0 indicates perfect performance. The model determined lower FEV_1_% predicted and FEV_1_/FVC, and higher FEF_25−75%_ % predicted and RDR_100_%/mg baseline variables as predictors of persistence of AHR to mannitol after 20 weeks daily treatment with ICS.

Univariate logistic regression analysis of baseline variables identified age, ICS treatment at baseline, FEV_1_, FEV_1_/FVC, FEF_25−75%_, PD_15_, and RDR_100_ as possible predictors (*p* < 0.1) of persistent AHR on follow-up (Table 6). Increased baseline RDR_100_ and FEF_25−75%_ and lower baseline FEV_1_ and FEV_1_/FVC remained significant independent predictors in the final multivariate model (Table 6) for which the AUC was 0.85 (Figure 4). The optimal threshold, corresponding to an estimated probability of 21%, yielded a sensitivity of 76%, specificity of 87%, a positive predictive value (PPV) of 53%, a negative predictive value (NPV) of 95%, and with a diagnostic odds ratio (DOR) of 21 to predict the likelihood of persistent AHR in a recruit after 20 weeks of daily ICS treatment. The estimated probability is calculated from baseline values of predictor variables from the logistic regression model as below:


$$\begin{array}{l} \text{Probability}=\text{ex}{\text{p}}^{\text{y}}/\left(1+\text{ex}{\text{p}}^{\text{y}}\right) \end{array}$$

where


$$\begin{array}{l} \text{y}=21.595-0.164\;*\;\left(\text{FE}{\text{V}}_{1}\text{\%pred}\right)-0.255\;*\;\left(\text{FE}{\text{V}}_{1}/\text{FVC\%}\right)\\ \;+0.138\;*\;\left(\text{FE}{\text{F}}_{25-75\text{\%}}\text{pred}\right)+0.70\;*\;\left(\text{RD}{\text{R}}_{100}\right) \end{array}$$

**Table 6:** Backward stepwise prediction model for persistent AHR on follow up.

**Baseline variable**	**Univariate OR (95%CI)**	***P***	**Multivariate adjusted OR (95%CI)**	***P***
Age, years	1.07 (1.0–1.14)	0.06		
PD_15_, mg	0.99 (0.99–1.0)	**0.001**		
ICS baseline	3.30 (1.34–8.13)	**<0.01**		
RDR_100_, %/mg	1.05 (1.02–1.08)	**<0.01**	1.07 (1.03–1.11)	**<0.001**
FEV_1_, %predicted	0.94 (0.90–0.98)	**<0.01**	0.85 (0.77–0.93)	**0.001**
FEV_1_/FVC%	0.93 (0.88–0.99)	**0.01**	0.78 (0.67–0.90)	**0.001**
FEF_25−75%_, %predicted	0.98 (0.95–1.0)	**0.04**	1.15 (1.06–1.25)	**0.001**

*Univariate and multivariate binary logistic regression of baseline variables. Bold values that indicate statistical significance*.

### Treatment Response in Moderate-Severe AHR to Mannitol (PD_15_ < 155 mg)

The group of recruits with moderate-severe AHR at baseline [PD_15_ <155 mg, *n* = 61/155, 39%] made up 72% of those with persistent AHR. Their spirometry measurements were significantly lower than the group with mild AHR to mannitol. Within the moderate-severe group in those in whom AHR persisted, baseline AHR was more severe and spirometry variables were lower than in those where AHR was abolished (Table 7). In those where AHR persisted, a greater proportion had an abnormal FEF_25−75%_ and an FEV_1_/FVC below the LLN. They were also more likely to be using ICS at the baseline visit and had a longer follow-up duration [50 days (95%CI 11–89), p < 0.05] (Table 7).

**Table 7:** Moderate to Severe AHR (PD_15_ < 155 mg) at baseline; demographics and baseline lung function, AHR and ICS treatment, type, and duration.

**Demographics**	**Airway sensitivity on follow up (PD**_**15**_ **)**			***P***
	**Persistent PD15**	**Abolished PD15**	**Difference (95%CI)**	
Total n	18	43		
Age, years, mean (SD)	24 ± 9	21 ± 4	3 (0.2 to 7)	**<0.05**
Males, n (%)	16 (89)	32 (74)		
Spirometry				
FEV_1_, % predicted, mean (SD)	86 ± 11	94 ± 10	8 (2 to 13)	**<0.01**
FEV_1_/FVC%, mean (SD)	72 ± 7	79 ± 7	7 (3 to 11)	**<0.01**
FEF_25−75%_, % predicted, mean (SD)	62 ± 14	76 ± 19	14 (4 to 24)	**<0.01**
Baseline FEF_25−75%_ <65% predicted, n (%)	12 (67)	13 (30)	37 (7 to 66)	**0.02**
Baseline FEV_1_/FVC <LLN, *n* (%)	9 (50)	8 (19)	31 (2 to 61)	**0.03**
AHR to mannitol				
PD_15_, mg, Gmean (95%CI)	53 (35 to 8 0)	86 (73 to 101)		**<0.01**
PD_15_, fold increase, Gmean (95%CI)	3.3 (2.3 to 4.7)	7.4 (6.3 to 8.7)		**<0.001**
PD_15_, doubling dose	1.7 (1.2 to 2.2)	2.9 (2.7 to 3.1)	1.2 (0.7 to 1.7)	**<0.001**
RDR_100_, %/mg, Gmean (95%CI)	24.3 (16.3 to 36.3)	16.3 (13.9 to 19.2)		**0.03**
RDR_100_, fold decrease, Gmean (95%CI)	1.7 (1.2 to 2.2)	4.0 (3.6 to 4.4)	2.4 (1.7 to 3.1)	**<0.001**
ICS Treatment				
ICS use at baseline, *n* (%)	9 (50)	6 (14)		**<0.01**
ICS alone, *n* (%)	16 (89)	36 (84)		0.71
Follow duration, days, mean (SD)	187 ± 90	137 ± 60	50 (11 to 89)	**<0.05**

*Values are mean ± SD or number (% of group). χ^2^ analysis used for proportions; Fisher exact used where estimated counts <5. PD_15_ and RDR_100_ Geometric mean (Gmean) 95%CI, logged for analysis. Bold values that indicate statistical significance*.

## Discussion

In this population of occupational recruits with a past or current history of asthma and AHR to mannitol, AHR was abolished in the majority following approximately 4 months of daily treatment with ICS. Even in this population with overall normal lung function, small but significant improvements in spirometry were still observed. Improvements in spirometry were weakly associated with the attenuation of AHR to mannitol. In a small proportion of the study population, AHR persisted following daily ICS treatment. However, this group achieved a modest improvement in responsiveness to mannitol while still having significant AHR (PD_15_ <635 mg) suggesting active EIB would still be present. This group with persistent AHR had more severe airway sensitivity (lower PD_15_) and reactivity (higher RDR_100_) to mannitol at baseline than those in whom AHR was abolished at follow-up. They also had significantly lower lung function at baseline; however, spirometry parameters did not improve. Further, this group was more likely to already be using ICS at the baseline assessment and had a longer follow-up duration, hence a longer treatment period. With daily ICS treatment, approximately one-third of recruits achieved no AHR (i.e., a flat dose response curve) and this airway reactivity resolved similar to what is seen in normal non-asthmatic subjects who have no AHR. Those that achieved a flat dose response curve with ICS treatment had the mildest AHR severity and most normal lung function at baseline.

Airway hyperresponsiveness to mannitol has been shown to be a useful screening tool used in occupational assessments of those at risk of asthma (8, 9). It has been demonstrated in people with mild asthma symptoms but no clinical asthma diagnosis. AHR to mannitol was 1.4 times more likely to identify AHR compared to a laboratory exercise test (19). Underreporting of asthma symptoms is common in similar occupational cohorts (8, 9). Therefore, the use of a reliable objective marker is preferable to the use of other more accessible clinical indicators of disease activity, such as symptoms and spirometry. The identification and subsequent resolution of AHR with ICS treatment enable the enlistment of persons with a history of asthma, reducing the risks of EIB during their employment.

These results, in a population being assessed for asthma or EIB as part of an occupational assessment, have replicated findings in prior clinical trials that have assessed the effectiveness of ICS on AHR to mannitol in people with confirmed asthma (4, 6, 20). Furthermore, AHR to mannitol identifies those with active asthma that can be treated effectively with low daily doses of ICS (20, 21). Thus, the ADF policy requires recruits to be treated with ICS alone at a maximum dose equivalent to 200 μg/day of beclomethasone for a 3-month trial period before reassessment (22). We were not able to identify the actual dose of daily ICS for all patients. However, as the majority of our cohort were ADF recruits and on low doses of ICS, these data support the findings of the efficacy of low daily doses of ICS that are useful to attenuate AHR to mannitol (20, 21). Overall, the group studied were determined to have clinically mild or intermittent asthma during their medical assessments carried out by occupational physicians and their spirometry was normal to near normal. However, many still had demonstrable AHR to mannitol, a feature identifying those whose asthma was currently active who would benefit from treatment with ICS. While significant improvements in spirometry were seen, these changes alone would not necessarily represent a discernible clinical benefit due to the normality of the measurements at baseline. Consistent with previous studies, we found only a weak relationship between AHR to mannitol and spirometry prior to therapy, and between changes in airway reactivity and spirometry with ICS treatment (4, 6). These findings reinforce the limitations of using spirometry to monitor ICS. It is expected that over 90% of the overall improvement in airway calibre with the commencement of ICS occurs over the first 2 weeks of treatment in people with asthma who have AHR to an indirect test (23). However, these data also highlight that if the patient is identified as having AHR by an indirect test, they have active airway inflammation, and thus further and potentially meaningful improvements in airway calibre are possible following many weeks of daily treatment, even if baseline spirometry is normal.

While AHR to mannitol is useful at predicting clinical response to ICS, a small proportion of recruits were observed to have persistent AHR despite daily ICS treatment. We expected that adherence to ICS treatment would have been high in our cohort as they were motivated by the goal of recruitment into their desired occupation. However, poor adherence may still have been a factor for some of these recruits, and we could not objectively document adherence to ICS in this clinical setting. However, we investigated variables that may predict the likelihood of slower or reduced ICS responsiveness. The data suggests that a slower improvement in AHR to mannitol in response to daily ICS is more likely if there is evidence of more significant airway obstruction before treatment assessment, and AHR to mannitol is in the moderate to severe range. These findings suggest these patients may have more active airway inflammation and possibly established airway remodelling, considering the lack of observed improvement in airway calibre. In people with asthma who are not taking regular ICS, moderate to severe AHR to mannitol (≤155 mg) is associated with significant sputum eosinophilia (median 8%), compared to the absence of sputum eosinophils (median <2%) in those with mild AHR (>155 mg) (2, 17, 24). These findings suggests that those with moderate to severe AHR to mannitol have a greater inflammatory contribution to their airway pathology. This increased inflammation may contribute to the slow initial response to daily treatment with ICS observed in the recruits with more severe AHR.

The number of recruits with AHR (PD_15_) persisting in this group was small relative to those in whom AHR was abolished. These individuals may be less steroid responsive, as they had a longer treatment period and may potentially need more time on ICS to inhibit their AHR more effectively (6). However, compared to those in whom AHR was abolished, a larger proportion of those where AHR persisted were already taking ICS at the baseline visit, supporting the suggestion of a more established or more active disease. Considering this, they were also a group with a higher proportion with an abnormal FEF_25−75%_ and FEV_1_/FVC at baseline. This may indicate the presence of a more fixed component of airway obstruction where it was also observed there was no improvement in spirometry, compared to those in whom AHR was abolished.

While most recruits abolished AHR to mannitol (noPD_15_), approximately one-third (32%) of the recruits still had a PD_10_ on follow-up. A PD_10_ has been found to be associated with the presence of EIB (14). Therefore, recruits with a PD_10_ alone following ICS may still be at risk of EIB, though it is likely to be mild. To further reduce the risk of EIB, resolving the PD_10_ to mannitol through extending the monitoring period may be desirable and achievable in occupational recruits. Flattening the dose response curve and suppressing airway reactivity (i.e., RDR_100_ <1) to a level of responsiveness seen in people without asthma was achieved in over a third (36%) of recruits (16). Demonstrating no AHR to mannitol is achievable with daily ICS treatment and maybe a more desirable goal of treatment, as the risk of EIB while performing occupational duties would be greatly reduced (25).

We performed a logistic regression model that predicted the liklihood of the persistence of AHR in this predominately young adult male population. For spirometry variables, lower measurements for FEV_1_ and FEV_1_/FVC increased the likelihood of AHR persisting. Greater airway reactivity (higher RDR_100_) also increased the likelihood of AHR persisting. However, in the final multivariate model, a lower FEF_25−75%_ reduced the likelihood of AHR persisting. Importantly, the probability of this model predicting response would likely change depending on the pre-test probability of the population to be tested. This model should be investigated further to determine if it can be validated and extrapolated to other populations, such as those with active asthma. Such a predictive capacity may assist with choosing different treatment regimens to increase the success of resolving AHR in recruits.

The main limitation of this study is that the data were collected retrospectively. Retrospective studies can introduce bias and while like other uncontrolled studies investigating ICS on AHR to indirect stimuli (4, 6, 20, 21, 25) this remains a limitation. In addition, adherence to ICS was not monitored objectively and future studies could include more objective markers of daily ICS adherence using electronic adherence monitors. Furthermore we were not able to collect additional clinical information in these recruits such as asthma symptoms, asthma control, allergic status, disease duration or prior therapy. This would be important to obtain while considering that recruits may not be willing to fully admit symptom burden due to the desire to enter a chosen occupation (8, 9). However, this is a real-world study that has produced similar findings to previous clinical trials, validating findings in a large clinical population showing that AHR to mannitol can be attenuated and abolished with daily ICS treatment (4, 6, 21). Further studies are needed to investigate if routine monitoring of ICS using AHR to mannitol is useful to achieve sustained clinical benefits in asthma symptom control. Further, it would be useful to establish if the resolution of AHR to mannitol represents the attenuation of asthma pathophysiology. While the resolution of AHR to mannitol increases the likelihood of resolution of EIB, future studies may be useful to confirm this likelihood.

Airway hyperresponsiveness to mannitol can be abolished with 20 weeks of daily treatment with ICS, in recruits who are seeking employment in careers that require strenuous physical exertion. This was achieved in the majority with low daily doses of ICS alone without LABA. Reduced pre-treatment spirometry variables and increased airway reactivity to mannitol predict the likelihood of an incomplete or slower response to daily ICS. Assessing AHR to mannitol with daily ICS may provide a useful objective marker to identify clinically meaningful attenuation of airway inflammation.

## Data Availability Statement

The original contributions presented in the study are included in the article/supplementary material, further inquiries can be directed to the corresponding author.

## Ethics Statement

The studies involving human participants were reviewed and approved by Western Sydney and Sydney Local Health District Human Research Ethics Committees. Written informed consent for participation was not required for this study in accordance with the national legislation and the institutional requirements.

## Author Contributions

CL contributed to trial design, data collection, preparation and analysis, manuscript preparation, and review. KW contributed to trial design, oversight of analysis, manuscript preparation, and review. CP contributed to data collection and manuscript review. HK contributed to trial design and manuscript review. JB contributed by supervising all aspects of the research, including the study concept and trial design, oversight of analysis, and manuscript preparation and review. All authors contributed to the article and approved the submitted version.

## Conflict of Interest

JB receives a 10% share of royalties for the sale of Aridol after distribution to Royal Prince Alfred Hospital in countries outside Australia and owns shares in Pharmaxis Ltd. CP owns shares in Pharmaxis Ltd. The remaining authors declare that the research was conducted in the absence of any commercial or financial relationships that could be construed as a potential conflict of interest.

## Publisher's Note

All claims expressed in this article are solely those of the authors and do not necessarily represent those of their affiliated organizations, or those of the publisher, the editors and the reviewers. Any product that may be evaluated in this article, or claim that may be made by its manufacturer, is not guaranteed or endorsed by the publisher.

## Abbreviations

ADF: Australian Defence Force
AHR: airway hyperresponsiveness
AUC: Area under the curve
BPT: Bronchial Provocation Test
EIB: Exercise induced bronchoconstriction
ICS: Inhaled Corticosteroids
LABA: Long-acting beta agonists
LLN: Lower limit of normal
NSWPF: New South Wales Police Force
PD_10_: Cumulative inhaled dose of mannitol to cause 10% fall in FEV_1_
PD_15_: Cumulative inhaled dose of mannitol to cause 15% fall in FEV_1_
PFL: Pulmonary Function Laboratories
ROC: Receiver operator characteristic
RDR: Response dose ratio.
